# Supplementary material for: Coordinated multiplexing of information about separate objects in visual cortex
Source: eLife. 2022 Nov 29;11:e76452. doi: 10.7554/eLife.76452 (PMC9708082; doi:10.7554/eLife.76452)
Supplement: Figure 4—source data 1. — The top two rows show the median spike count correlations observed for dual stimuli for various types of pairs of units, and correspond to the data shown in Figure 4 and Figure 5 in the main text (gray background). The next three rows show the same analyses conducted for trials involving single stimuli. Here, the ‘congruent’ group was subdivided according to whether the presented stimulus was the one that elicited the stronger response (‘driven’) or the weaker one (‘not driven’). The bottom two rows show the differences in the medians observed for the relevant congruent and incongruent groups (lines 1 minus 2 and lines 3 minus 5; green background). [file elife-76452-fig4-data1.docx]

|  | **All** | **Mixture-Mixture pairs** | **Intermediate - Intermediate pairs** | **Single-Single pairs** |
| --- | --- | --- | --- | --- |
| **Congruent AB** | 0.252 | 0.486 | 0.263 | 0.233 |
| **Incongruent AB** | -0.052 | -0.140 | -0.009 | -0.032 |
| **Congruent driven A or B** | 0.251 | 0.384 | 0.266 | 0.250 |
| **Congruent not driven A or B** | 0.145 | 0.132 | 0.142 | 0.147 |
| **Incongruent A or B** | 0.116 | 0.127 | 0.076 | 0.108 |
|  |  |  |  |  |
| **Congruent minus Incongruent AB** | 0.304 | 0.626 | 0.272 | 0.265 |
| **Congruent driven minus incongruent A or B** | 0.135 | 0.257 | 0.190 | 0.142 |

Figure 4 - Supplementary Table. Median spike count correlations for additional subgroups of the V1 adjacent stimuli dataset. The top two rows show the median spike count correlations observed for dual stimuli for various types of pairs of units, and correspond to the data shown in figures 4 and 5 in the main text (gray background). The next three rows show the same analyses conducted for trials involving single stimuli. Here, the “congruent” group was subdivided according to whether the presented stimulus was the one that elicited the stronger response (“driven”) or the weaker one (“not driven”). The bottom two rows show the differences in the medians observed for the relevant congruent and incongruent groups (lines 1 minus 2 and lines 3 minus 5; green background).
