# Supplementary figures and images for: Coordinated multiplexing of information about separate objects in visual cortex
Source: eLife. 2022 Nov 29;11:e76452. doi: 10.7554/eLife.76452 (PMC9708082; doi:10.7554/eLife.76452)

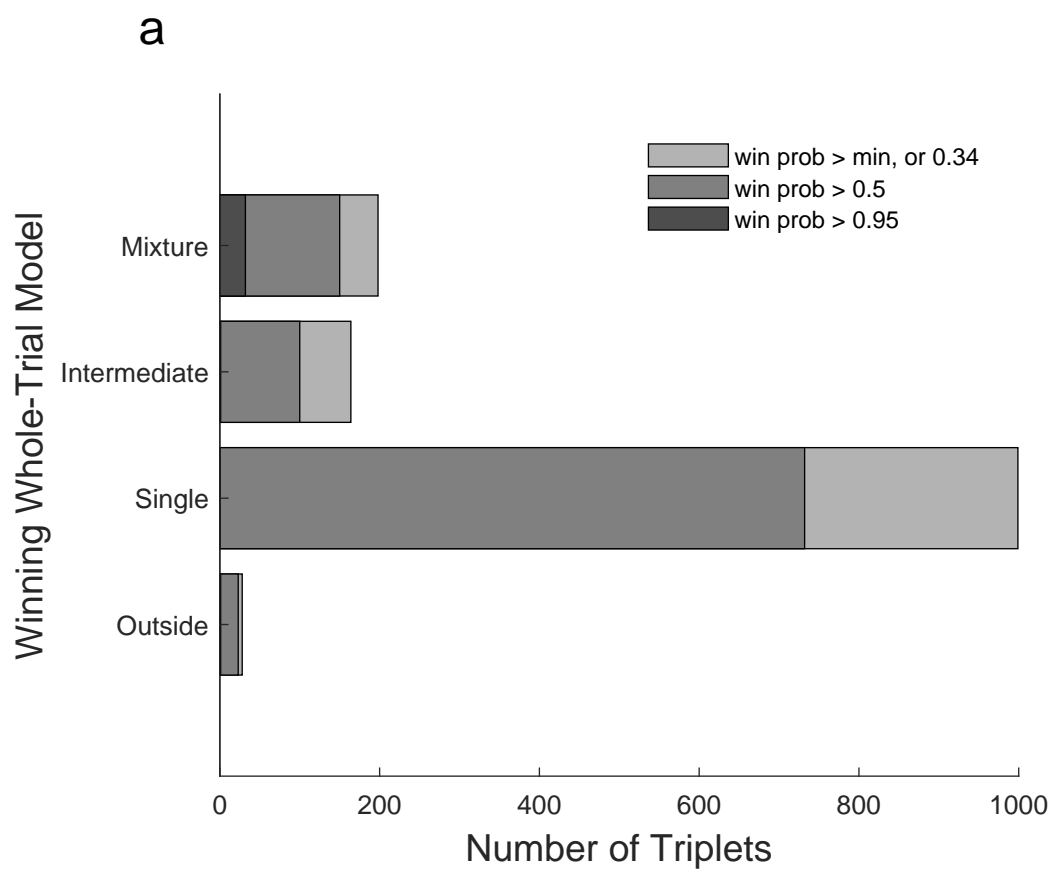

Supplement: Source data 1. — The file and folder names are informative regarding which analyses they relate to. Some analyses are based on multiple runs of the modeling code, with slight variations due to the probabilistic nature of the analysis. [file elife-76452-data1.zip › SourceData2022/plots_and_outputs/adjacent_v1.pdf]

**N = 306 1083**

WinPr > 0.67

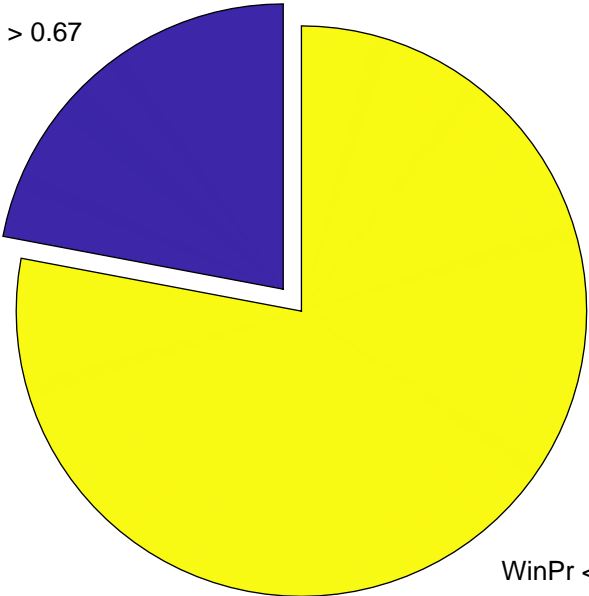

WinPr < 0.67

Supplement: Source data 1. — The file and folder names are informative regarding which analyses they relate to. Some analyses are based on multiple runs of the modeling code, with slight variations due to the probabilistic nature of the analysis. [file elife-76452-data1.zip › SourceData2022/plots_and_outputs/adjacent_v1.pdf_pie.pdf]

a

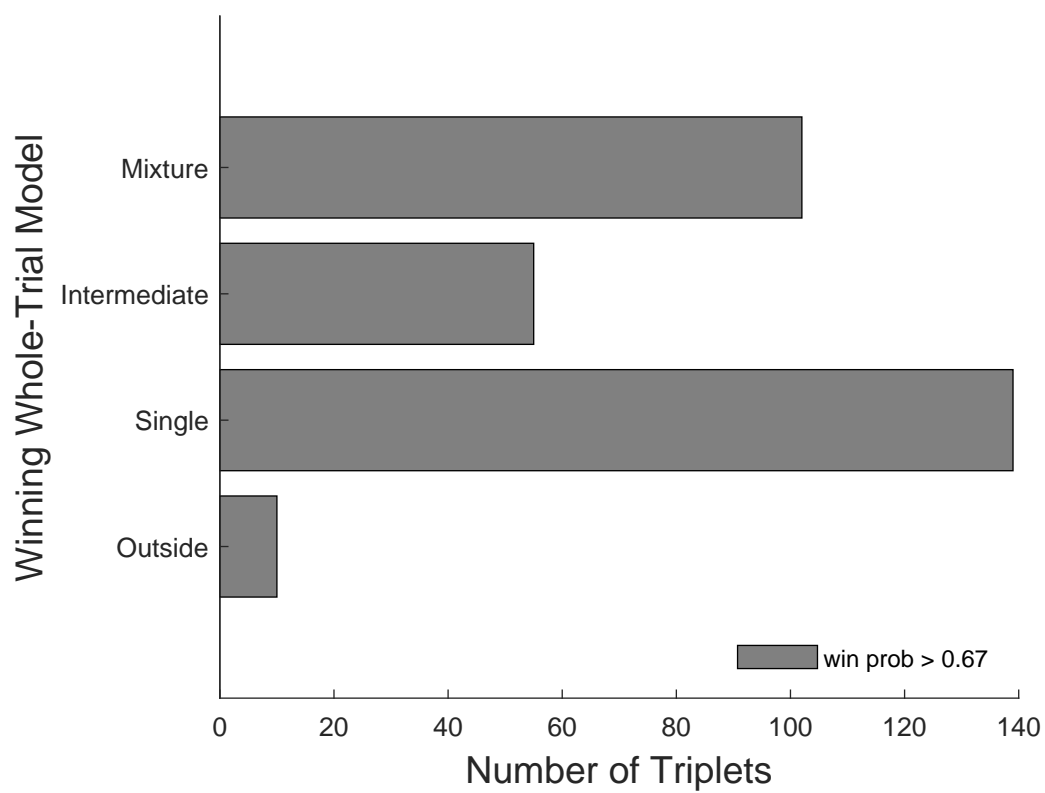

Supplement: Source data 1. — The file and folder names are informative regarding which analyses they relate to. Some analyses are based on multiple runs of the modeling code, with slight variations due to the probabilistic nature of the analysis. [file elife-76452-data1.zip › SourceData2022/plots_and_outputs/adjacent_v1.pdf_singlecrit.pdf]

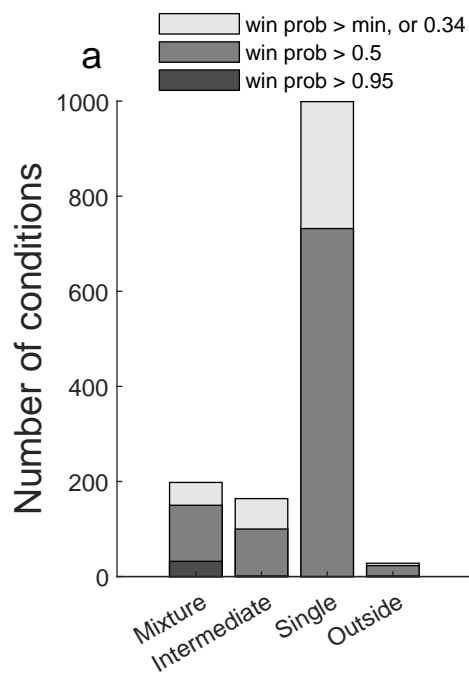

Winning Whole-Trial Model

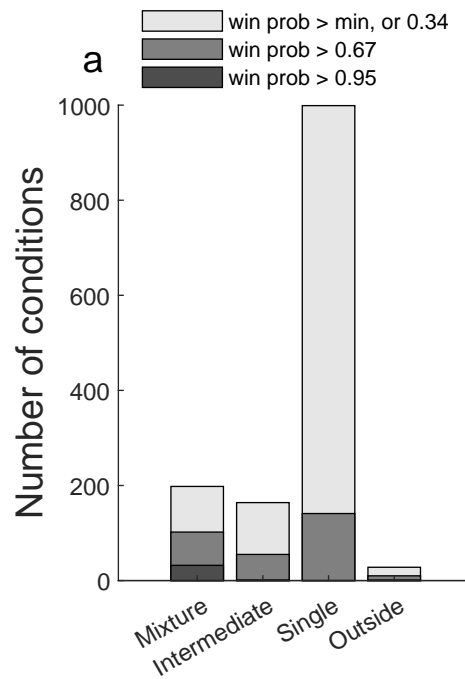

Winning Whole-Trial Model

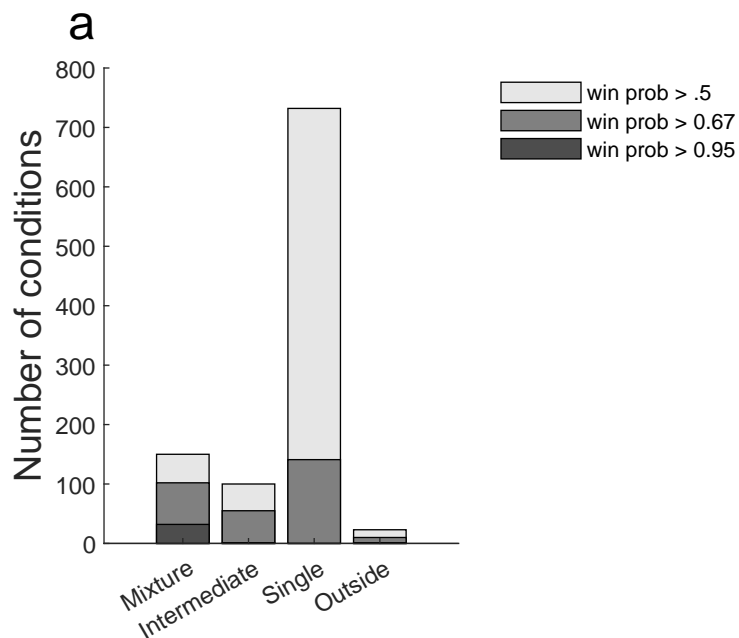

Winning Whole-Trial Model

Supplement: Source data 1. — The file and folder names are informative regarding which analyses they relate to. Some analyses are based on multiple runs of the modeling code, with slight variations due to the probabilistic nature of the analysis. [file elife-76452-data1.zip › SourceData2022/plots_and_outputs/adjacent_v1.pdf_vert]

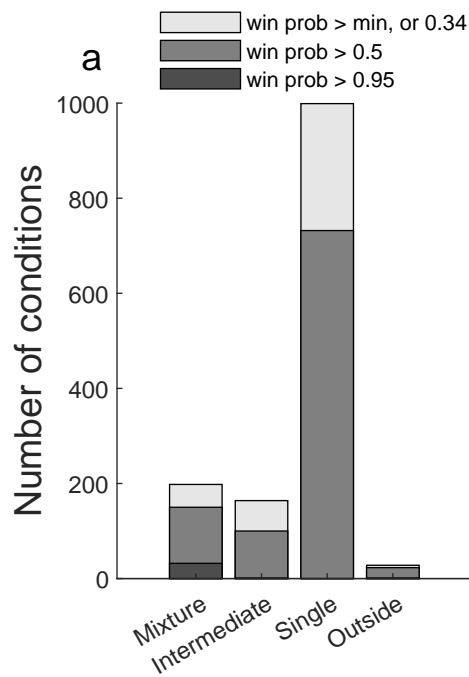

Winning Whole-Trial Model

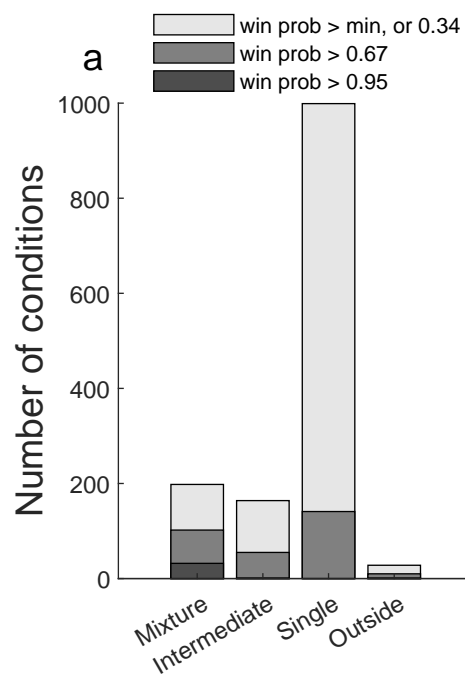

Winning Whole-Trial Model

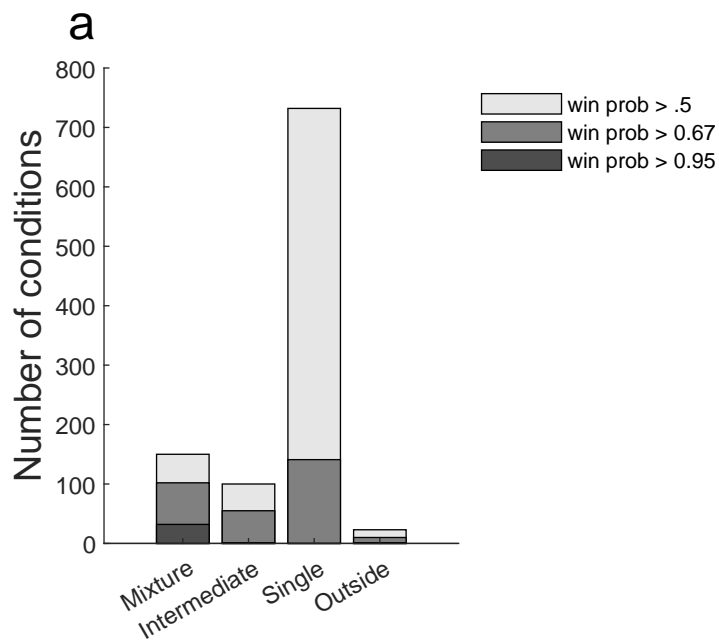

Winning Whole-Trial Model

Supplement: Source data 1. — The file and folder names are informative regarding which analyses they relate to. Some analyses are based on multiple runs of the modeling code, with slight variations due to the probabilistic nature of the analysis. [file elife-76452-data1.zip › SourceData2022/plots_and_outputs/adjacent_v1.pdf_vert.pdf]

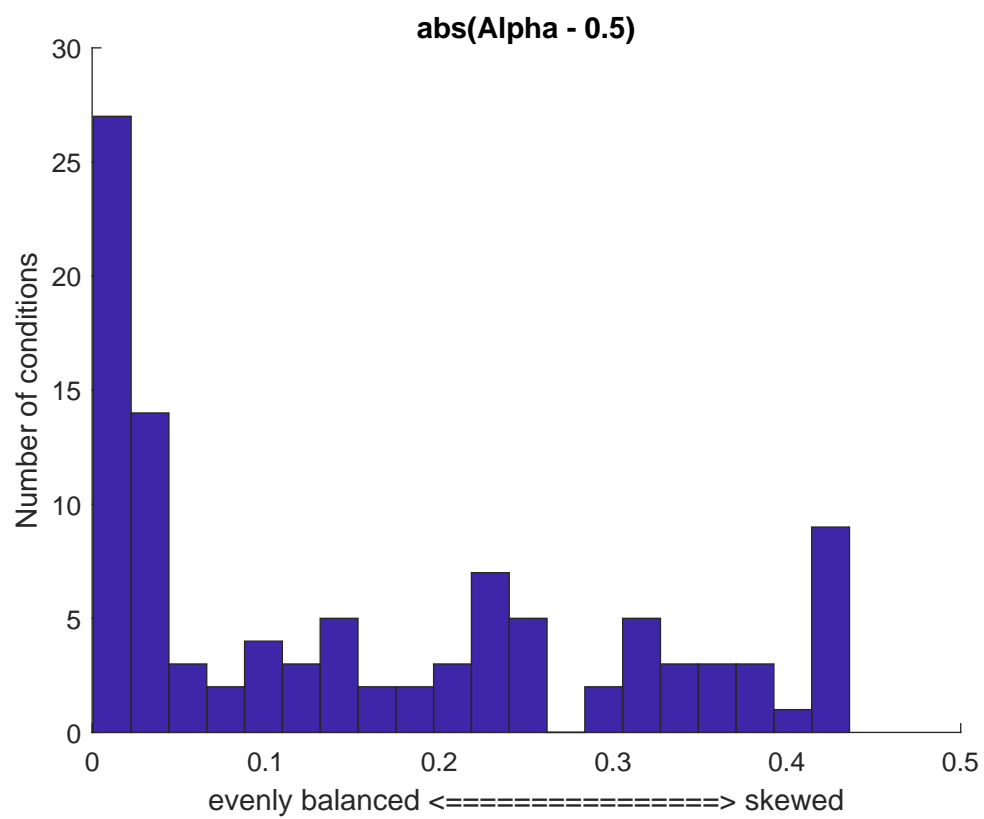

Supplement: Source data 1. — The file and folder names are informative regarding which analyses they relate to. Some analyses are based on multiple runs of the modeling code, with slight variations due to the probabilistic nature of the analysis. [file elife-76452-data1.zip › SourceData2022/plots_and_outputs/alpha_distributions.pdf]

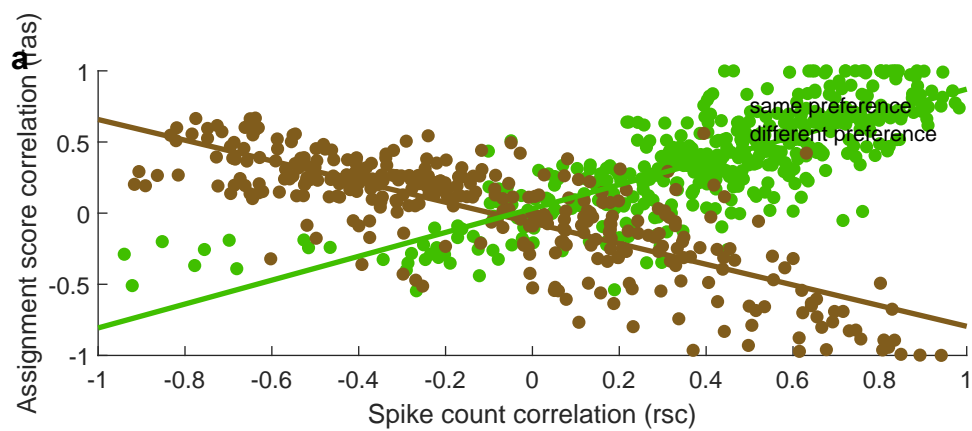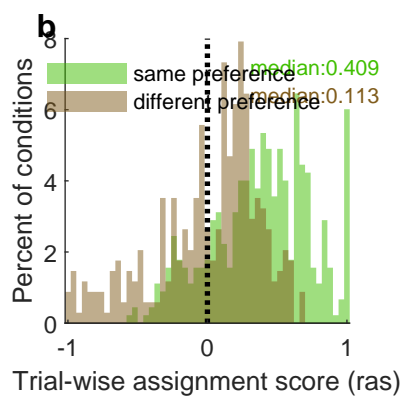

Supplement: Source data 1. — The file and folder names are informative regarding which analyses they relate to. Some analyses are based on multiple runs of the modeling code, with slight variations due to the probabilistic nature of the analysis. [file elife-76452-data1.zip › SourceData2022/plots_and_outputs/assignment_scoreMay2022_latency.pdf]

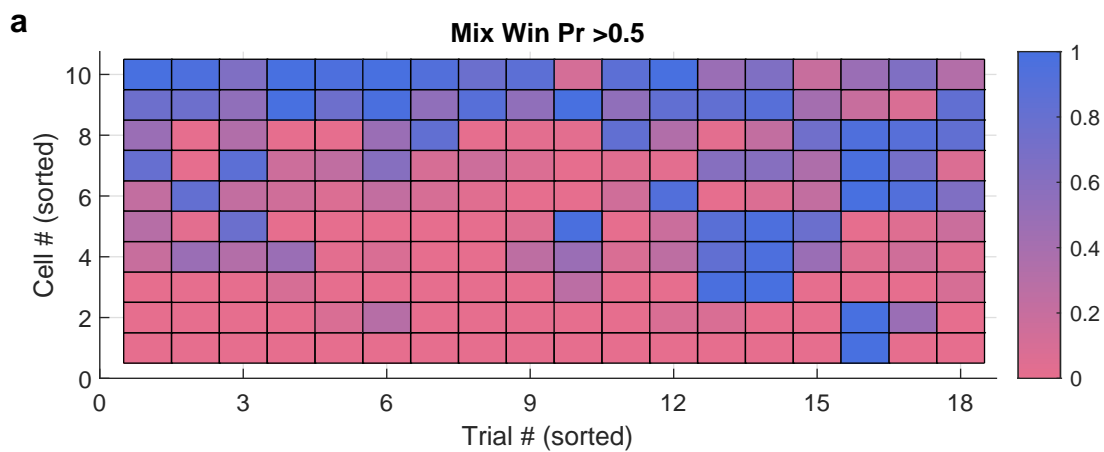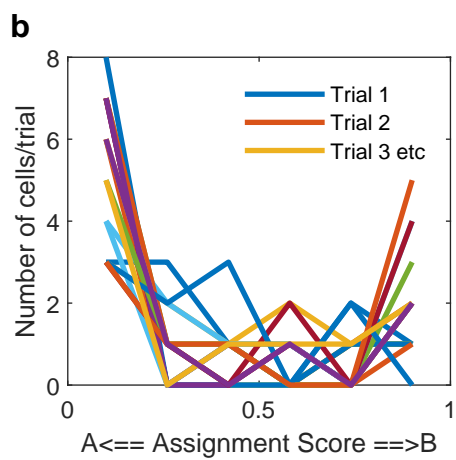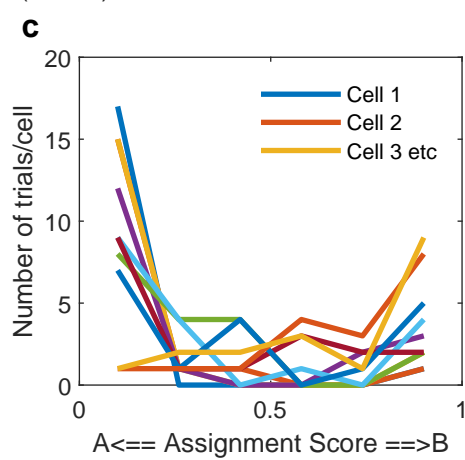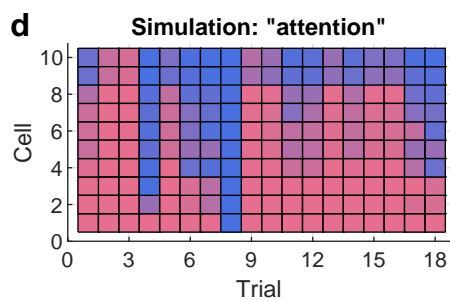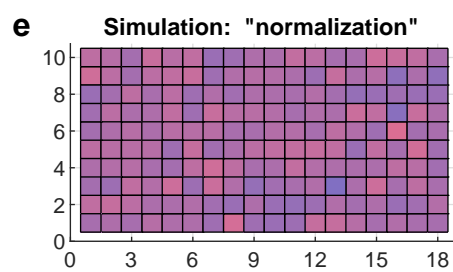

Supplement: Source data 1. — The file and folder names are informative regarding which analyses they relate to. Some analyses are based on multiple runs of the modeling code, with slight variations due to the probabilistic nature of the analysis. [file elife-76452-data1.zip › SourceData2022/plots_and_outputs/assignment_score_population_by_trial.pdf]

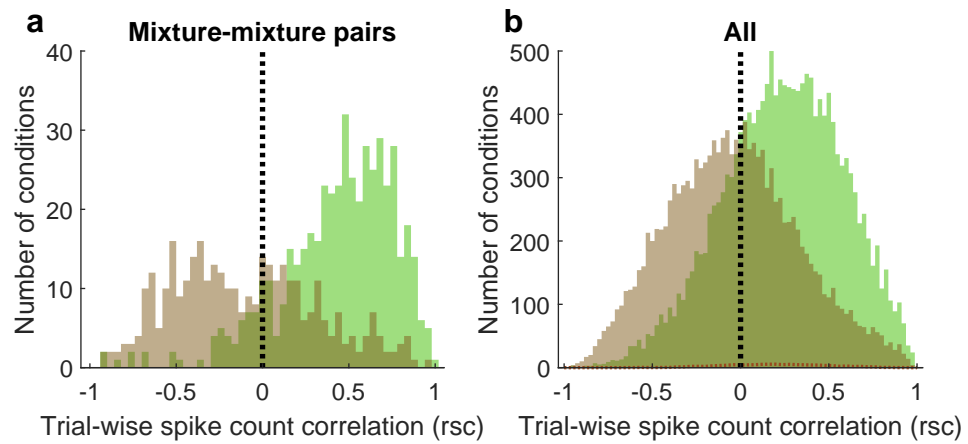

Supplement: Source data 1. — The file and folder names are informative regarding which analyses they relate to. Some analyses are based on multiple runs of the modeling code, with slight variations due to the probabilistic nature of the analysis. [file elife-76452-data1.zip › SourceData2022/plots_and_outputs/non_normalized_same_diff.pdf]

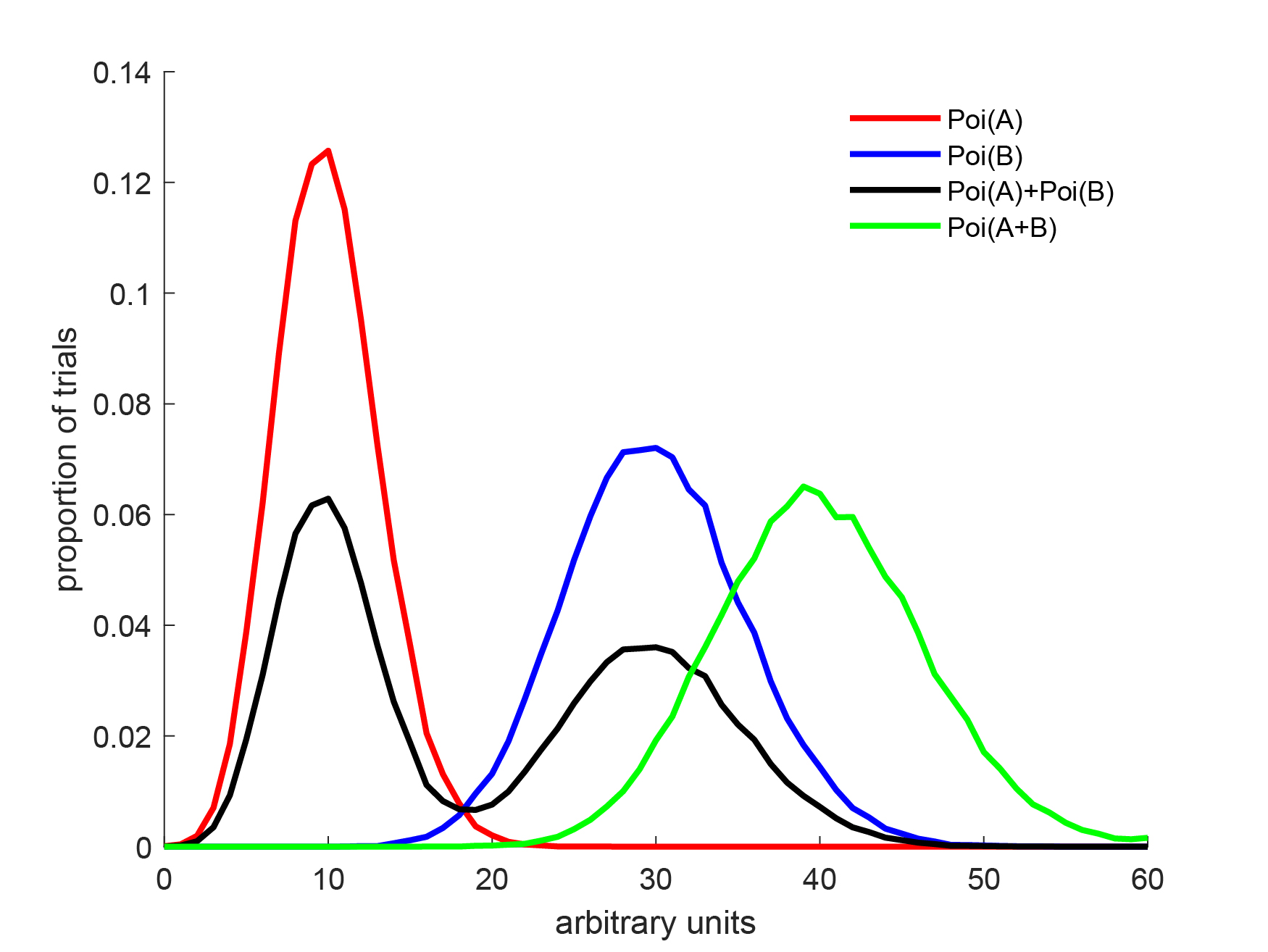

Supplement: Source data 1. — The file and folder names are informative regarding which analyses they relate to. Some analyses are based on multiple runs of the modeling code, with slight variations due to the probabilistic nature of the analysis. [file elife-76452-data1.zip › SourceData2022/plots_and_outputs/poisson_for_reviewers.jpg]

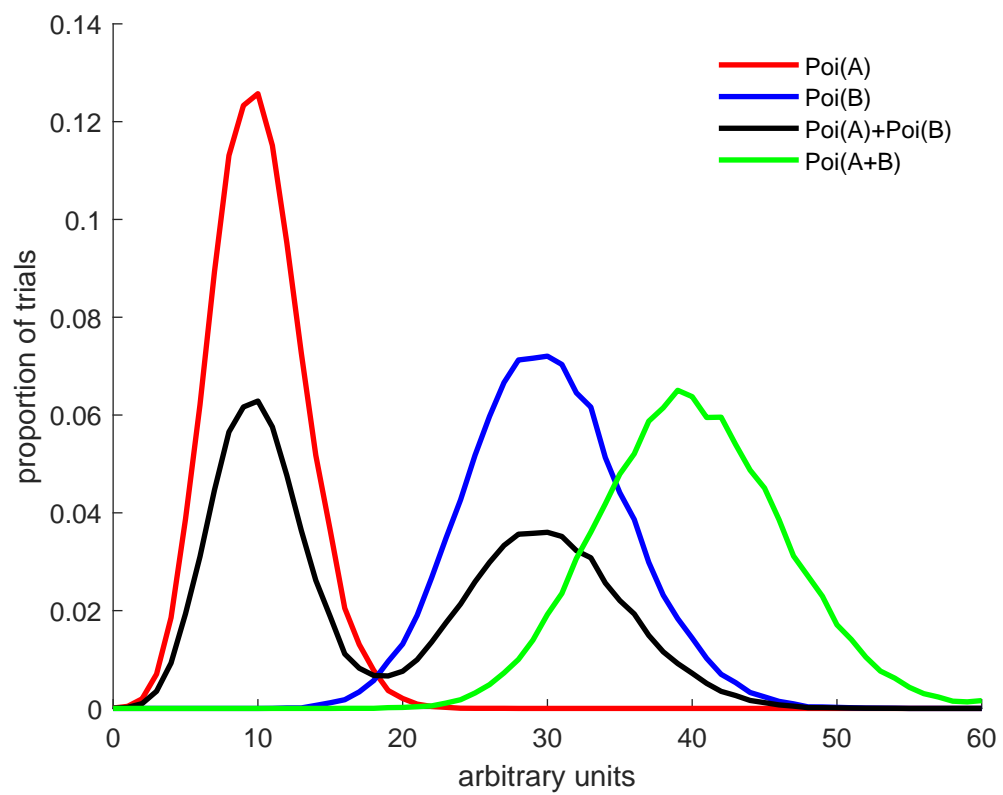

Supplement: Source data 1. — The file and folder names are informative regarding which analyses they relate to. Some analyses are based on multiple runs of the modeling code, with slight variations due to the probabilistic nature of the analysis. [file elife-76452-data1.zip › SourceData2022/plots_and_outputs/poisson_for_reviewers.pdf]

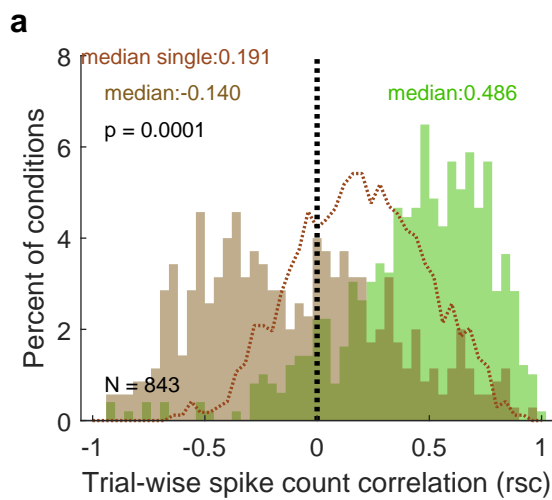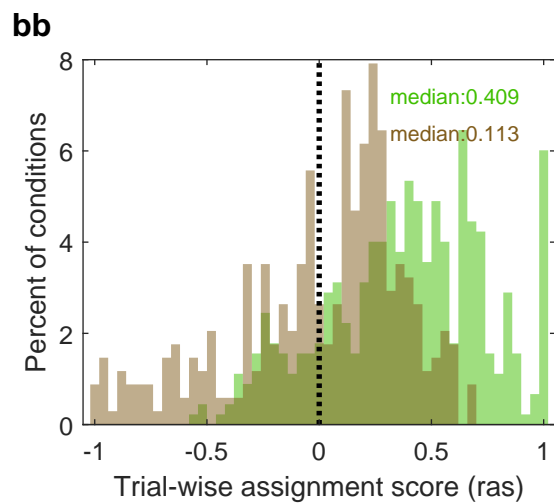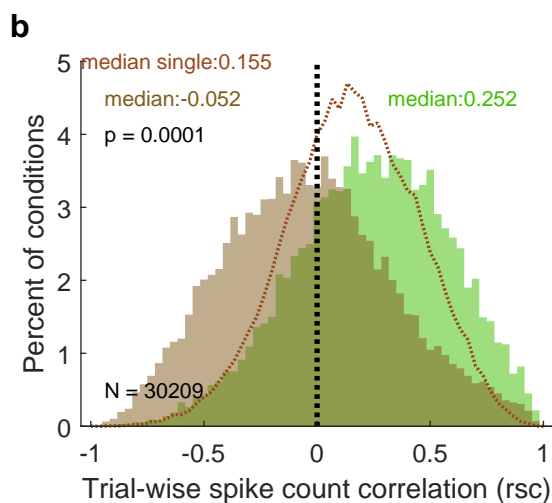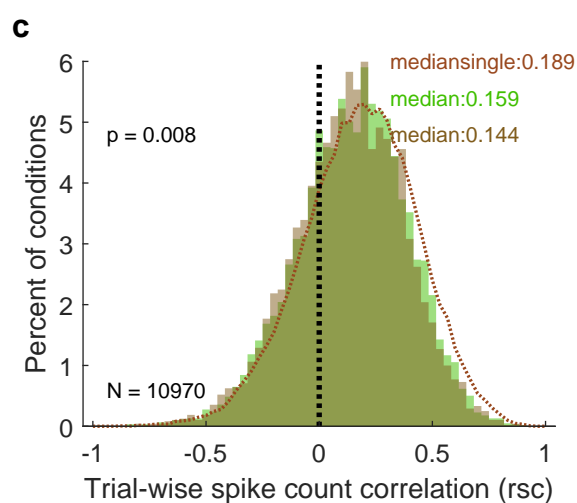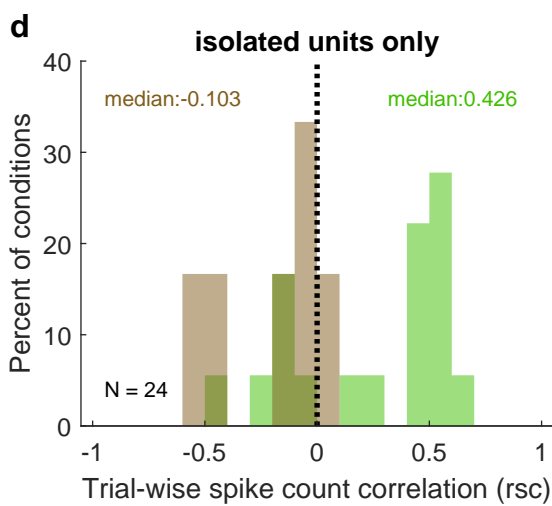

same preference  
different preference  
singles

Supplement: Source data 1. — The file and folder names are informative regarding which analyses they relate to. Some analyses are based on multiple runs of the modeling code, with slight variations due to the probabilistic nature of the analysis. [file elife-76452-data1.zip › SourceData2022/plots_and_outputs/results_figure_with_mixtures_rearrange_May2022_latency.pdf]

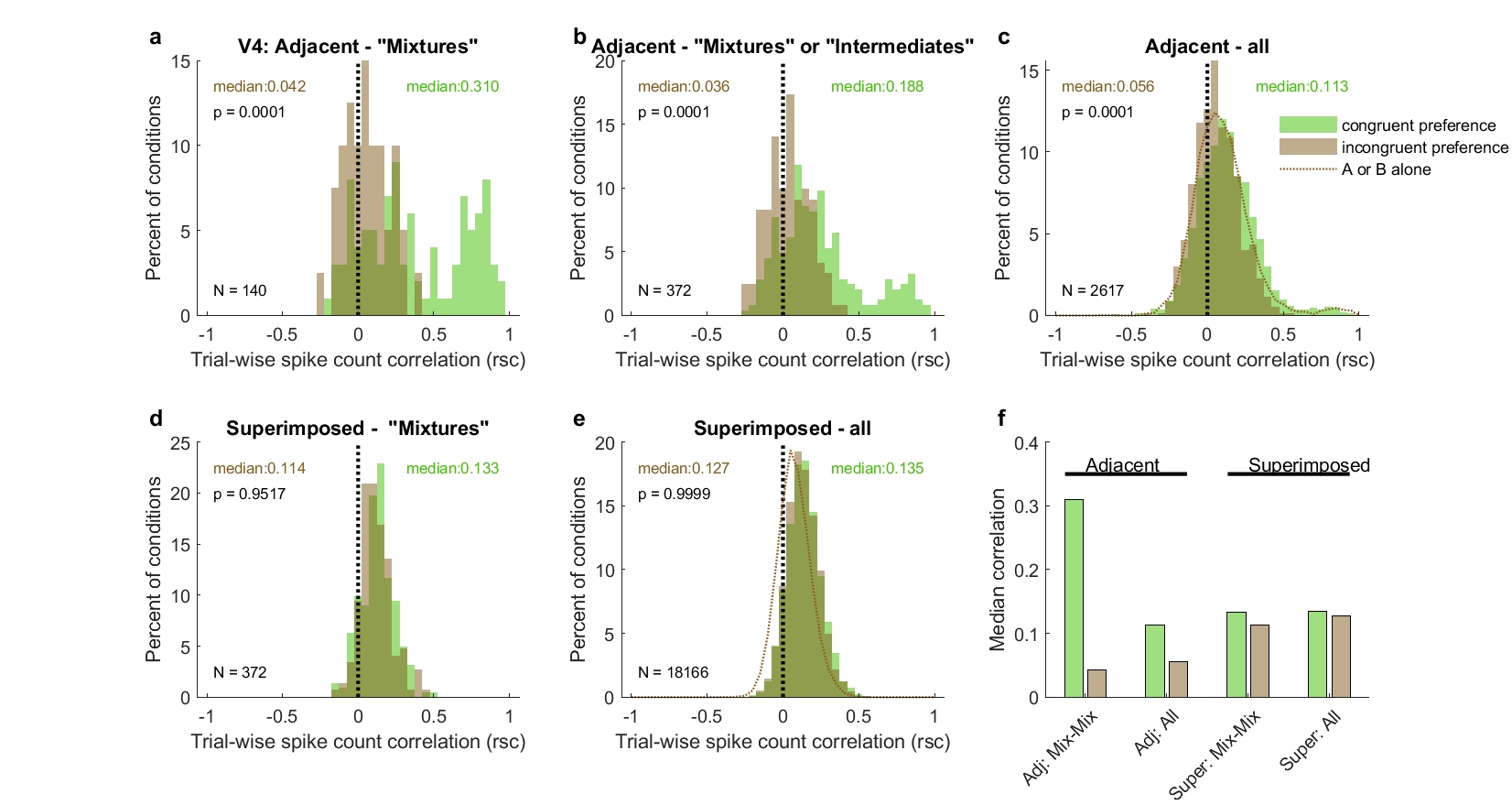

Supplement: Source data 1. — The file and folder names are informative regarding which analyses they relate to. Some analyses are based on multiple runs of the modeling code, with slight variations due to the probabilistic nature of the analysis. [file elife-76452-data1.zip › SourceData2022/plots_and_outputs/results_with_mixtures_V4_rsc.jpg]

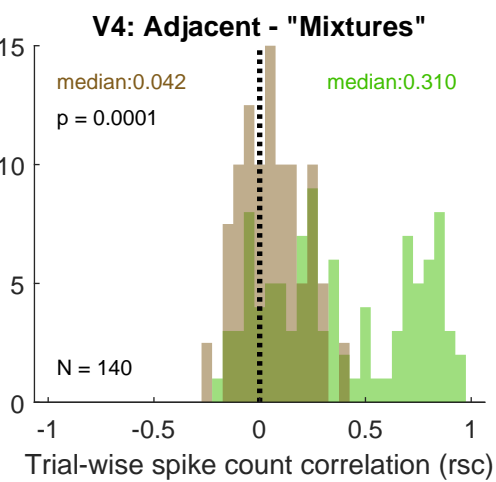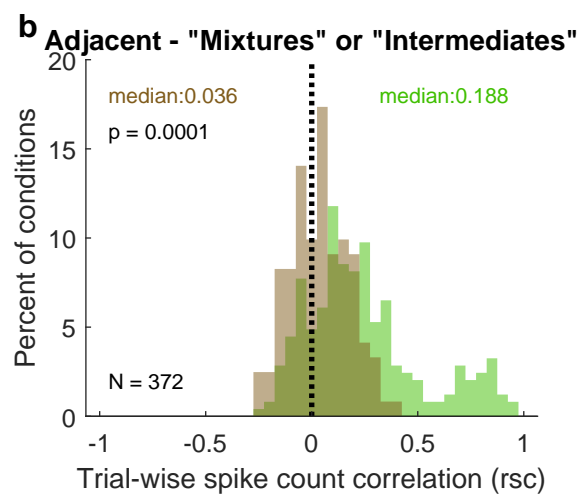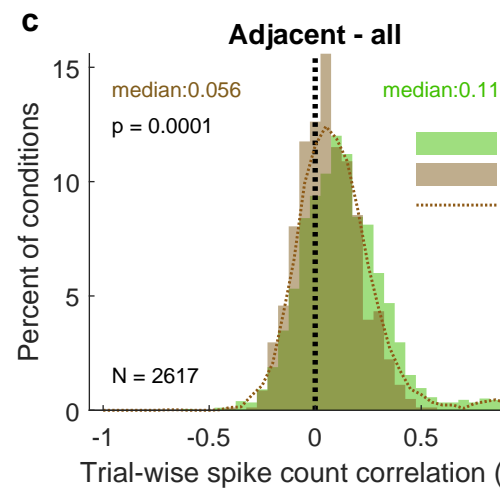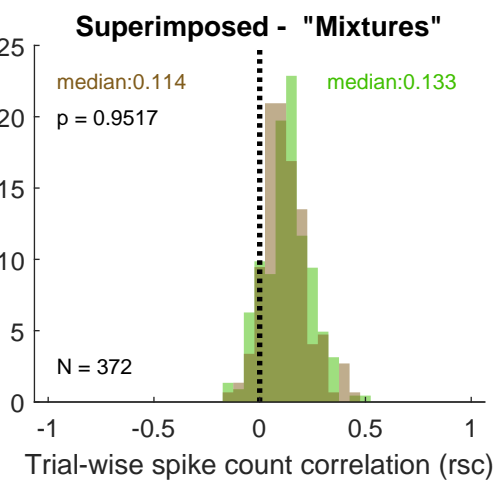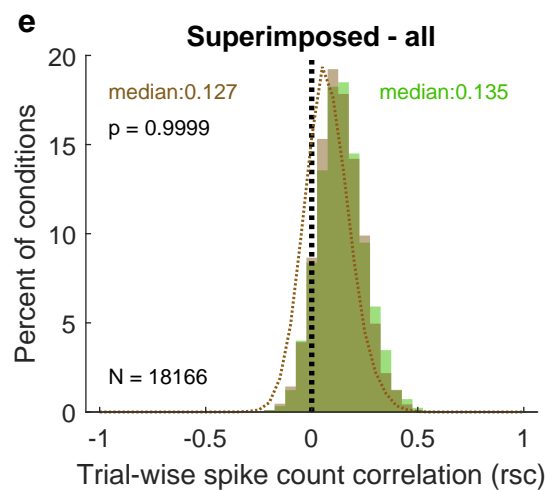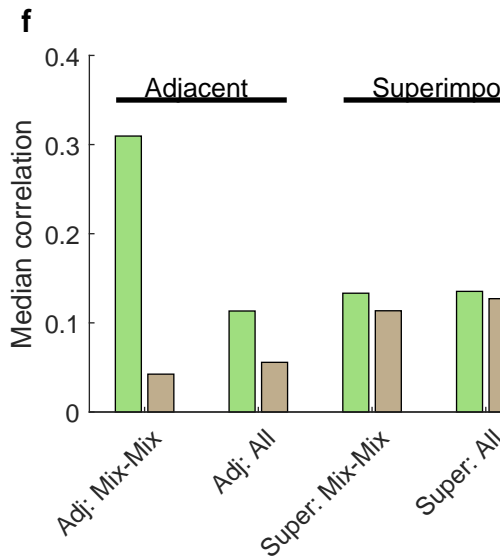

Supplement: Source data 1. — The file and folder names are informative regarding which analyses they relate to. Some analyses are based on multiple runs of the modeling code, with slight variations due to the probabilistic nature of the analysis. [file elife-76452-data1.zip › SourceData2022/plots_and_outputs/results_with_mixtures_V4_rsc_gabor_and_image.pdf]

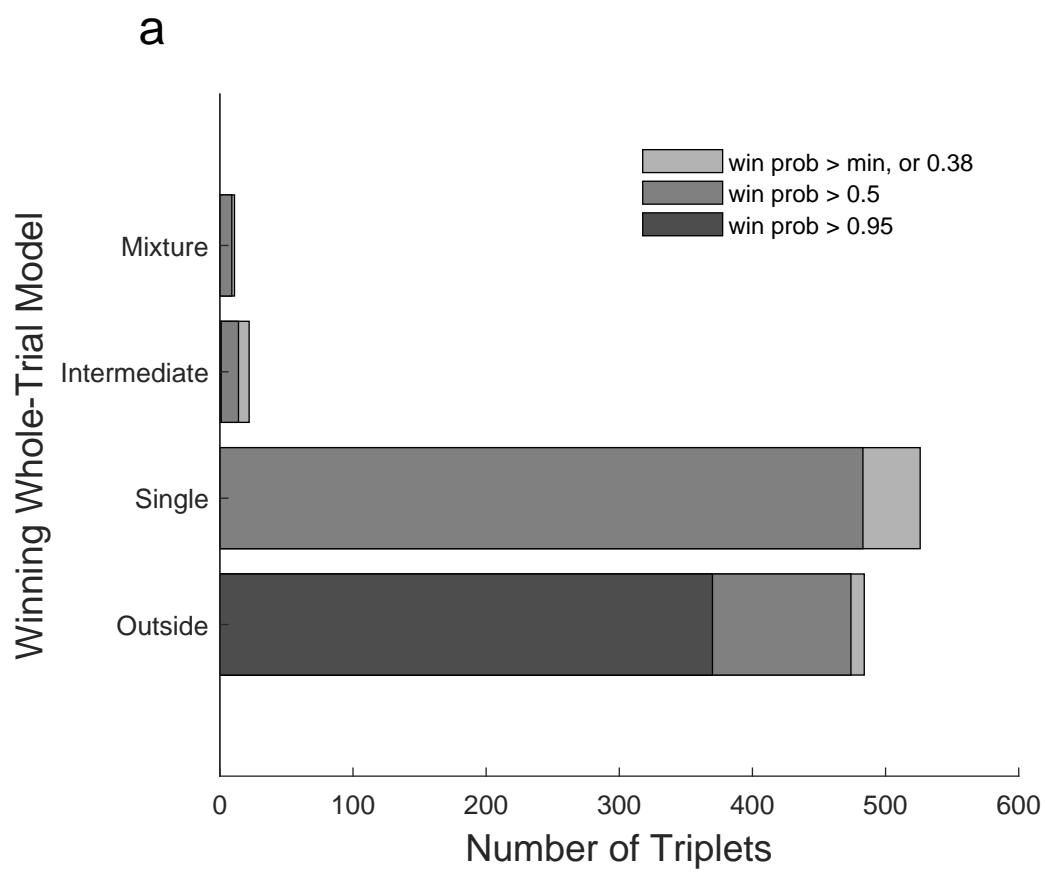

Supplement: Source data 1. — The file and folder names are informative regarding which analyses they relate to. Some analyses are based on multiple runs of the modeling code, with slight variations due to the probabilistic nature of the analysis. [file elife-76452-data1.zip › SourceData2022/plots_and_outputs/superimposed_v1_30_230.pdf]

**N = 667 376**

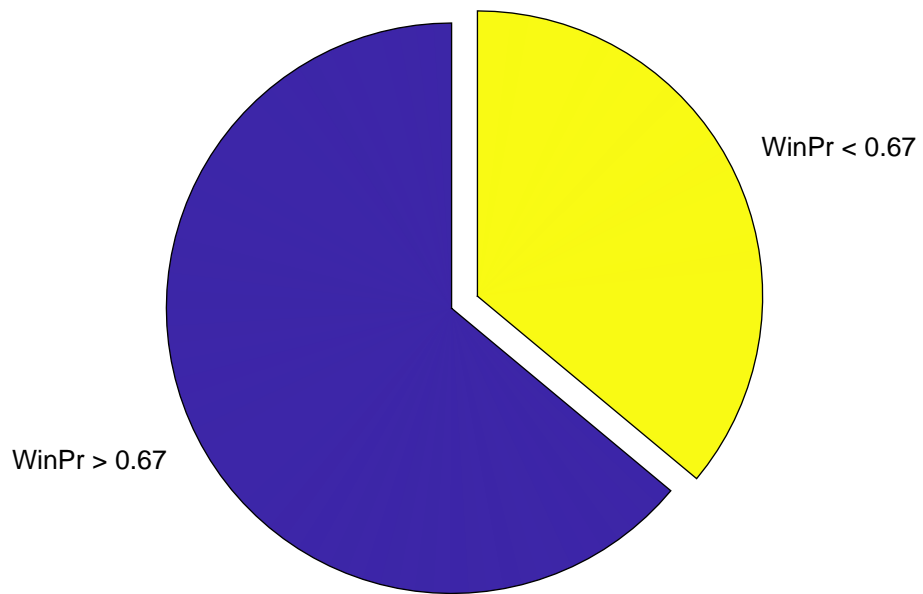

Supplement: Source data 1. — The file and folder names are informative regarding which analyses they relate to. Some analyses are based on multiple runs of the modeling code, with slight variations due to the probabilistic nature of the analysis. [file elife-76452-data1.zip › SourceData2022/plots_and_outputs/superimposed_v1_30_230.pdf_pie.pdf]

a

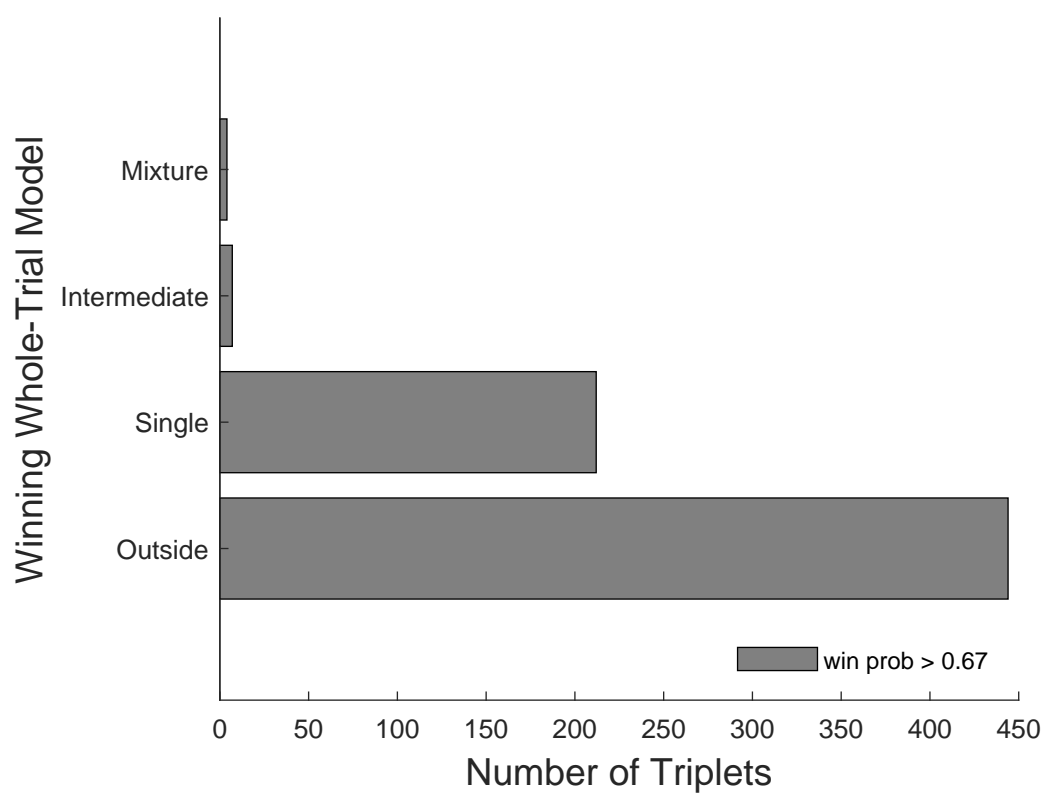

Supplement: Source data 1. — The file and folder names are informative regarding which analyses they relate to. Some analyses are based on multiple runs of the modeling code, with slight variations due to the probabilistic nature of the analysis. [file elife-76452-data1.zip › SourceData2022/plots_and_outputs/superimposed_v1_30_230.pdf_singlecrit.pdf]

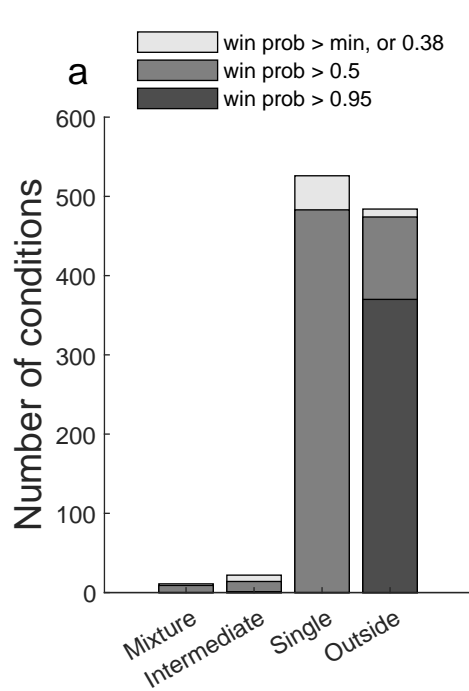

Winning Whole-Trial Model

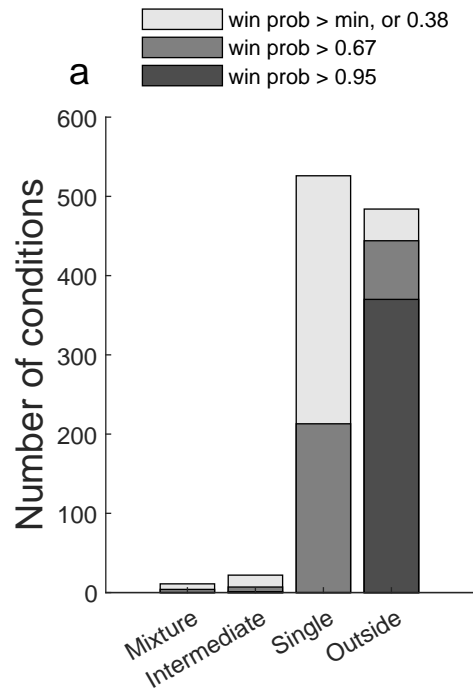

Winning Whole-Trial Model

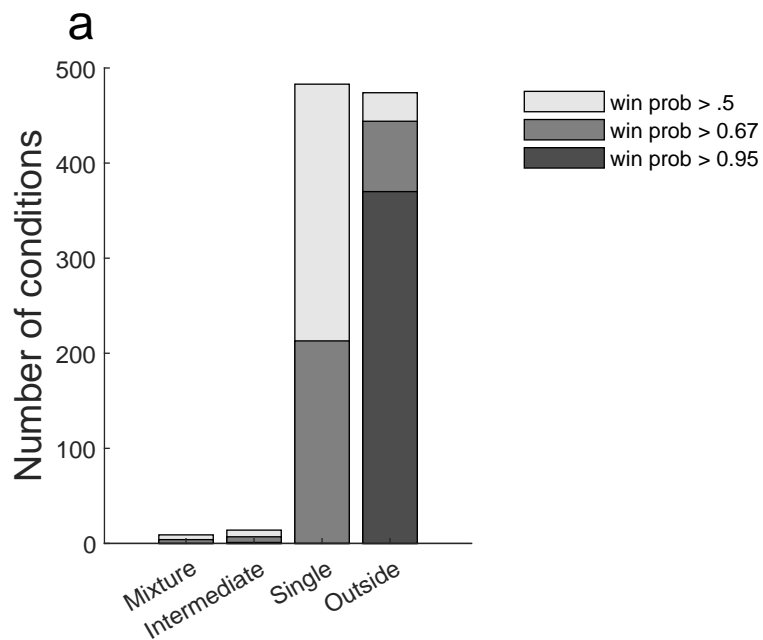

Winning Whole-Trial Model

Supplement: Source data 1. — The file and folder names are informative regarding which analyses they relate to. Some analyses are based on multiple runs of the modeling code, with slight variations due to the probabilistic nature of the analysis. [file elife-76452-data1.zip › SourceData2022/plots_and_outputs/superimposed_v1_30_230.pdf_vert.pdf]

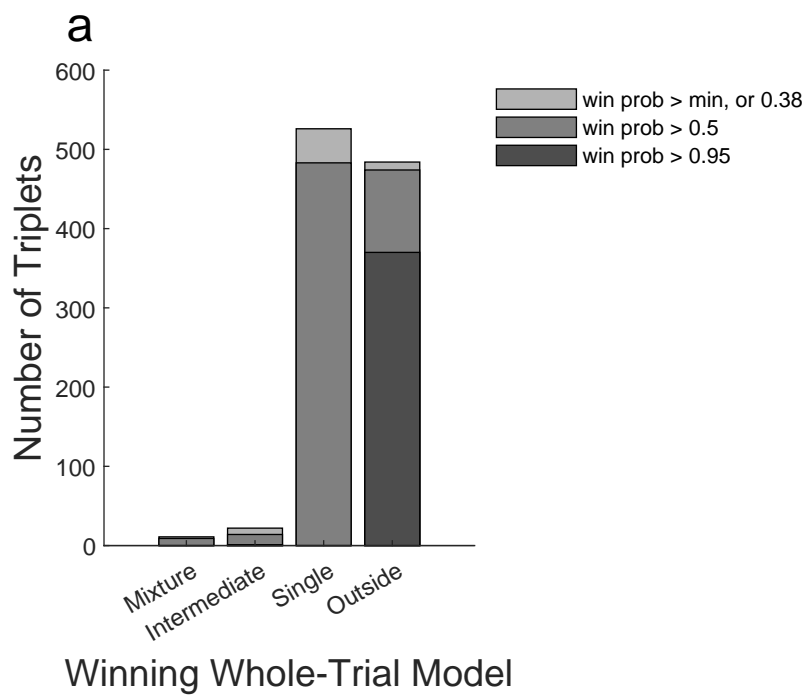

Supplement: Source data 1. — The file and folder names are informative regarding which analyses they relate to. Some analyses are based on multiple runs of the modeling code, with slight variations due to the probabilistic nature of the analysis. [file elife-76452-data1.zip › SourceData2022/plots_and_outputs/superimposed_v1_30_230_vert.pdf]

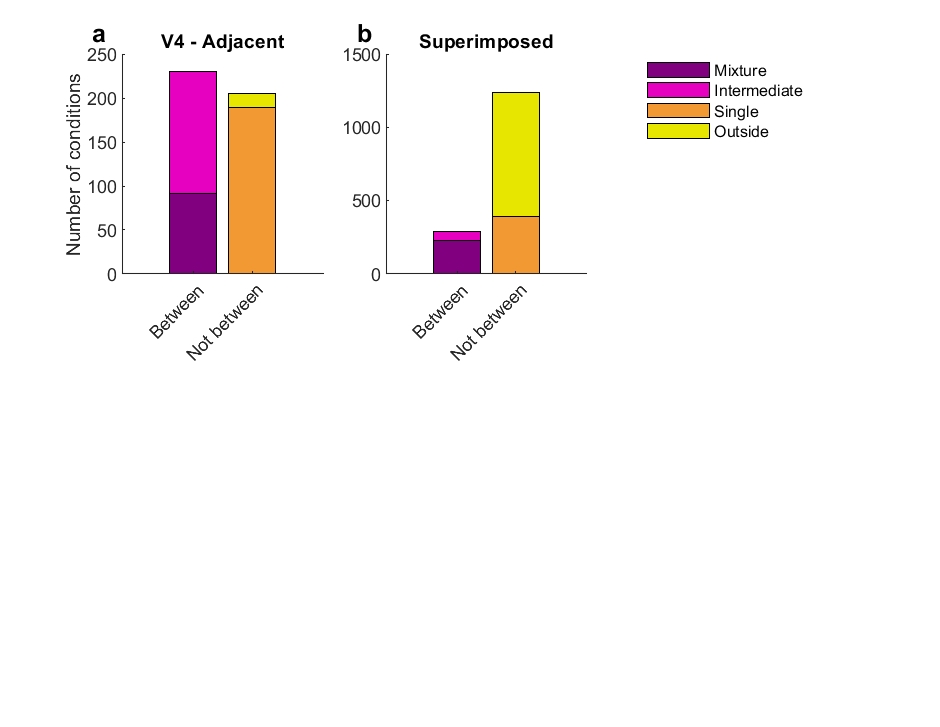

Supplement: Source data 1. — The file and folder names are informative regarding which analyses they relate to. Some analyses are based on multiple runs of the modeling code, with slight variations due to the probabilistic nature of the analysis. [file elife-76452-data1.zip › SourceData2022/plots_and_outputs/V4_whole_color_bar_plot_May2022.jpg]

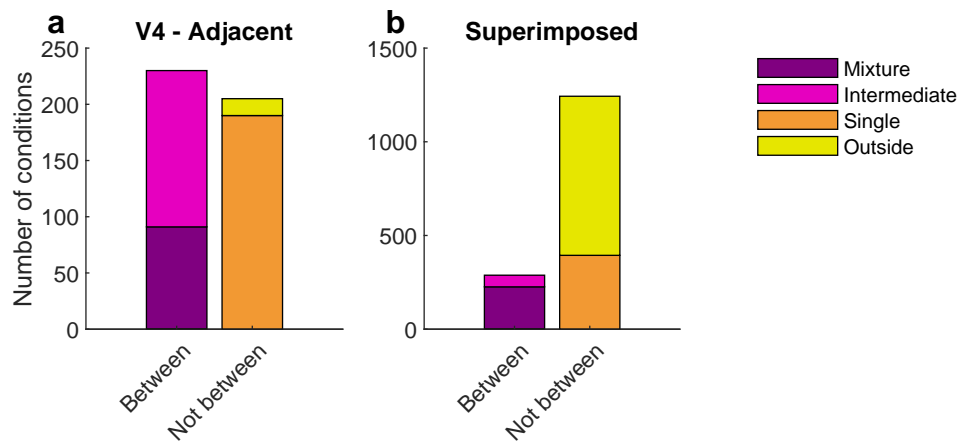

Supplement: Source data 1. — The file and folder names are informative regarding which analyses they relate to. Some analyses are based on multiple runs of the modeling code, with slight variations due to the probabilistic nature of the analysis. [file elife-76452-data1.zip › SourceData2022/plots_and_outputs/V4_whole_color_bar_plot_May2022.pdf]

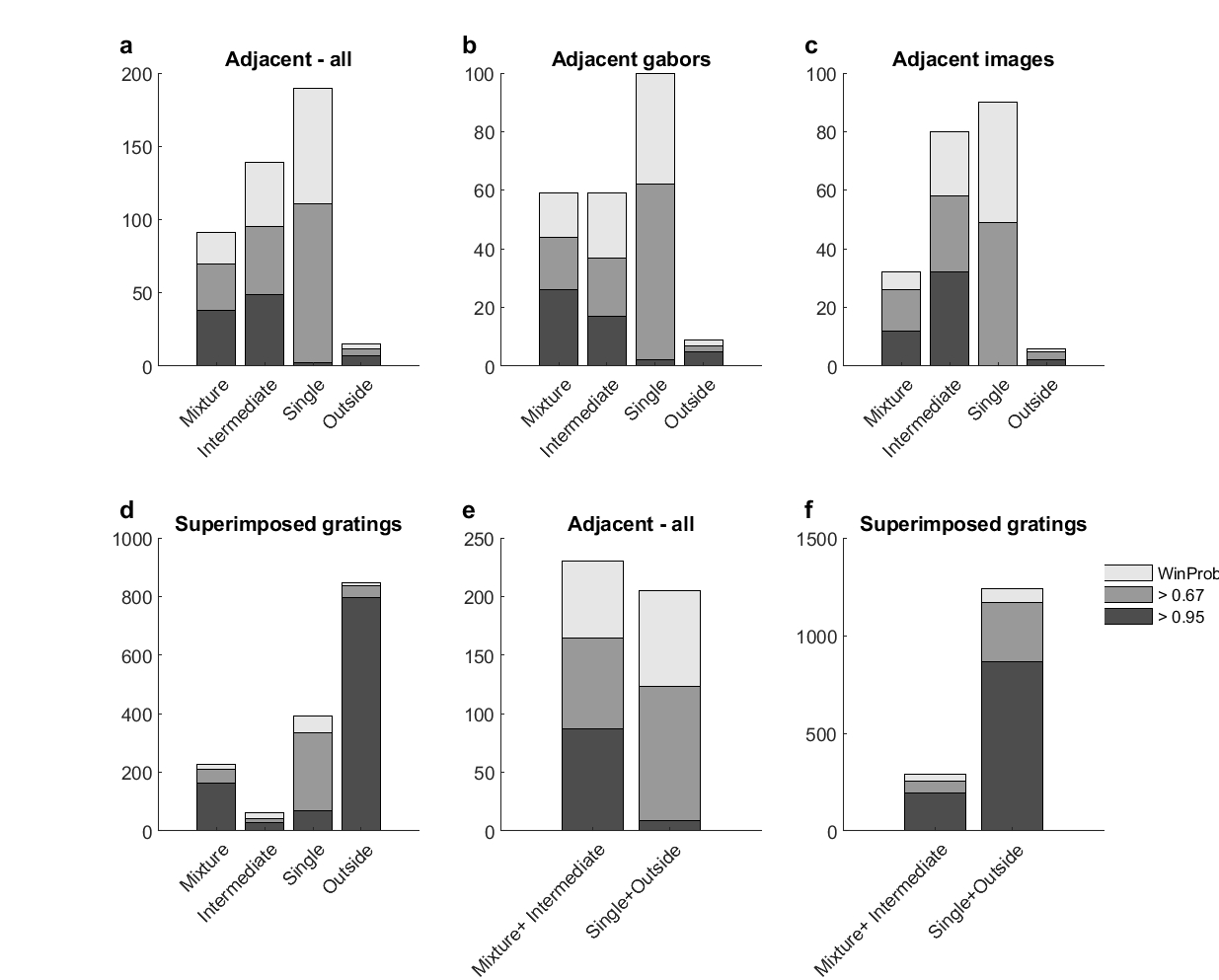

Supplement: Source data 1. — The file and folder names are informative regarding which analyses they relate to. Some analyses are based on multiple runs of the modeling code, with slight variations due to the probabilistic nature of the analysis. [file elife-76452-data1.zip › SourceData2022/plots_and_outputs/V4_whole_complete_May2022.jpg]

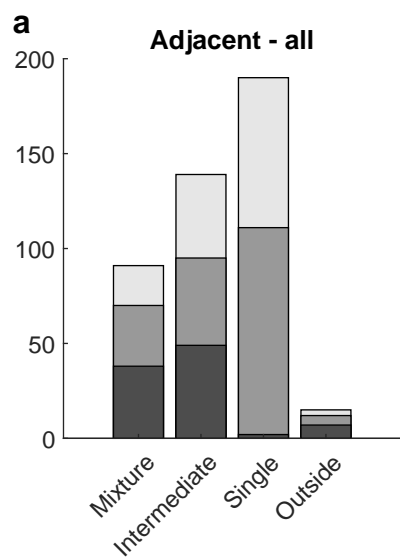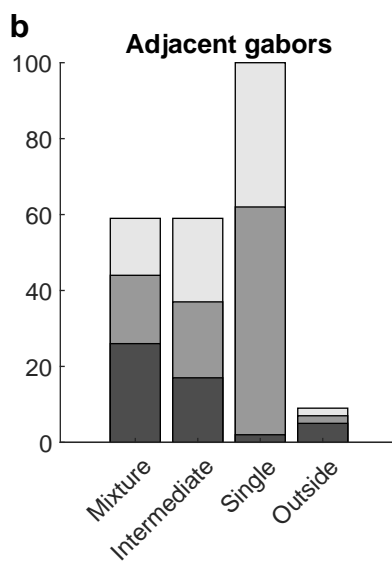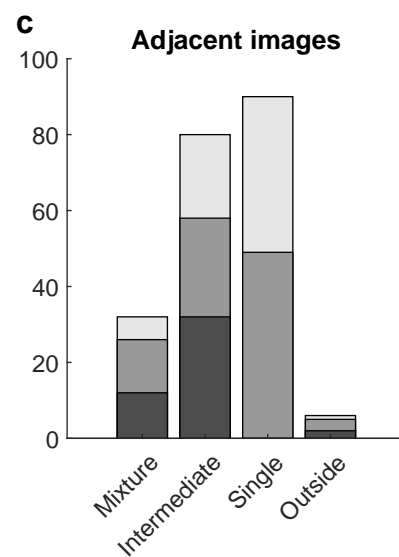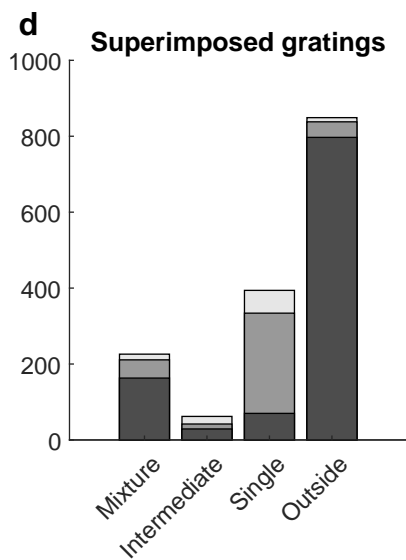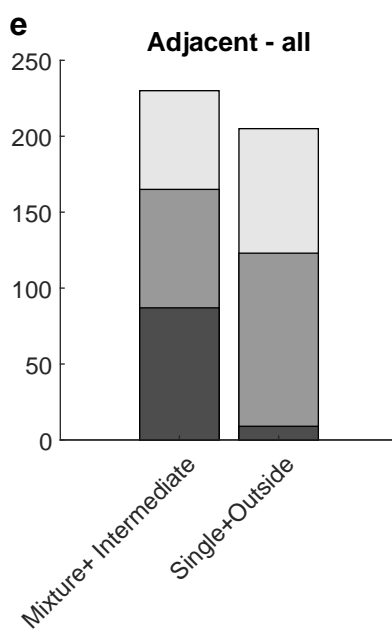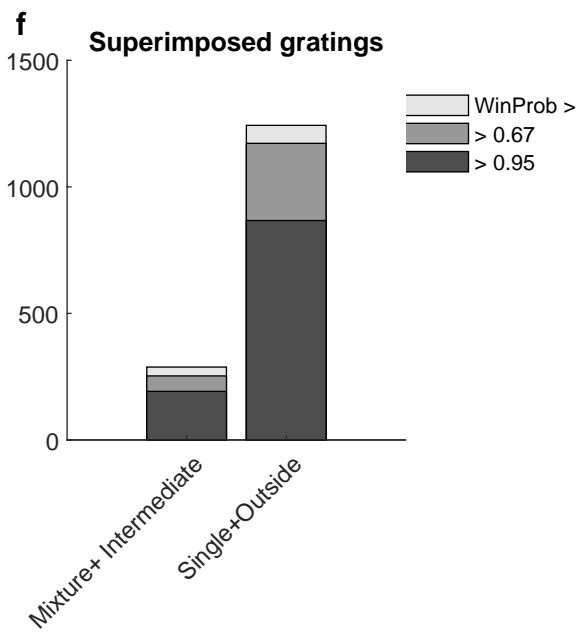

Supplement: Source data 1. — The file and folder names are informative regarding which analyses they relate to. Some analyses are based on multiple runs of the modeling code, with slight variations due to the probabilistic nature of the analysis. [file elife-76452-data1.zip › SourceData2022/plots_and_outputs/V4_whole_complete_May2022.pdf]
